# Melatonin Levels in Preterm and Term Infants and Their Mothers

**DOI:** 10.3390/ijms20092077

**Published:** 2019-04-27

**Authors:** Valérie Biran, Fabrice Decobert, Nathalie Bednarek, Priscilla Boizeau, Jean-François Benoist, Bruno Claustrat, Jérôme Barré, Marina Colella, Alice Frérot, Roselyne Garnotel, Olivier Graesslin, Bassam Haddad, Jean-Marie Launay, Thomas Schmitz, Julien Schroedt, Anne-Laure Virlouvet, Sophie Guilmin-Crépon, Adyla Yacoubi, Evelyne Jacqz-Aigrain, Pierre Gressens, Corinne Alberti, Olivier Baud

**Affiliations:** 1 Neonatal Intensive Care Unit, Assistance Publique-Hôpitaux de Paris, Robert Debré Children’s Hospital, University Paris Diderot, Sorbonne Paris-Cité, 75019 Paris, France; Olivier.BAUD@hcuge.ch; 2 PROTECT, Inserm 1141, Université Paris Diderot, Sorbonne Paris Cité, 75019 Paris, France; pierre.gressens@inserm.fr; 3 PremUP Foundation, 75014 Paris, France; fabrice.decobert@chicreteil.fr; 4 Neonatal Intensive Care Unit, Centre Hospitalier Intercommunal, 94010 Créteil, France; 5 Neonatal Intensive Care Unit, American Memorial Hospital, 51100 Reims, France; nbednarek@chu-reims.fr; 6 Unit of Clinical Epidemiology, Assistance Publique-Hôpitaux de Paris, Robert Debré Children’s Hospital, University Paris Diderot, Sorbonne Paris-Cité, Inserm U1123 and CIC-EC 1426, 75019 Paris, France; priscilla.boizeau@aphp.fr (P.B.); corinne.alberti@aphp.fr (C.A.); 7 Biochemistry Department, Assistance Publique-Hôpitaux de Paris, Robert Debré Children’s Hospital, 75019 Paris, France; jean-francois.benoist@aphp.fr; 8 Hormonology Department, Groupement hospitalier Est-Hospices Civils de Lyon, 69500 Bron, France; bruno.claustrat@chu-lyon.fr; 9 Centre de Ressources Biologiques, Centre Hospitalier Intercommunal Créteil, 94010 Créteil, France; jerome.barre@chicreteil.fr; 10 Neonatal Intensive Care Unit, Robert Debré Hospital, 75019 Paris, France; marina.colella@aphp.fr (M.C.); alice.frerot@aphp.fr (A.F.); anne-laure.virlouvet@aphp.fr (A.-L.V.); 11 Biochemistry Laboratory, American Memorial Hospital Reims, 51100 Reims, France; rgarnotel@chu-reims.fr; 12 Department of Gynecology and Obstetrics, American Memorial Hospital Reims, 51100 Reims, France; ograesslin@chu-reims.fr; 13 Department of Gynecology and Obstetrics, Centre Hospitalier Intercommunal Créteil, 94010 Créteil, France; bassam.haddad@chicreteil.fr; 14 Biochemistry and Molecular Laboratory, Lariboisière Hospital, 75019 Paris, France; jean-marie.launay@aphp.fr; 15 Department of Gynecology and Obstetrics, Robert Debré Hospital, 75019 Paris, France; thomas.schmitz@aphp.fr; 16 UEC CIC 1426, Robert Debré Hospital, 75019 Paris, France; julien.schroedt@aphp.fr (J.S.); sophie.guilmin-crepon@aphp.fr (S.G.-C.); adyla.yacoubi@aphp.fr (A.Y.); 17 Department of Pharmacology and Pharmacogenetics, Assistance Publique-Hôpitaux de Paris, Robert Debré Children’s Hospital, 75019 Paris, France; evelyne.jacqz-aigrain@aphp.fr; 18 Centre for the Developing Brain, Division of Imaging Sciences and Biomedical Engineering, King’s College London, King’s Health Partners, St. Thomas’ Hospital, London SE1 7EH, UK; 19 Division of Neonatology and Pediatric Intensive Care, Children’s University Hospital and University of Geneva, 1205 Geneva, Switzerland

**Keywords:** melatonin, prematurity, term infants, neuroprotection, brain development

## Abstract

The prevention of perinatal brain damage following preterm birth remains a public health priority. Melatonin has been shown to be a promising neuroprotectant in neonatal preclinical models of brain damage, but few studies have investigated melatonin secretion in newborns. We hypothesized that melatonin circulating levels would be lower in preterm compared to term infants. We conducted a prospective, longitudinal, multicenter study to assess melatonin, and 6-sulfatoxy-melatonin (aMT6s) concentrations, measured by radioimmunoassay. Among 209 neonates recruited, 110 were born before 34 gestational weeks (GW) and 99 born after 34 GW. Plasma melatonin concentrations, measured at birth and on Day 3 were below detectable levels (≤7 pg/mL) in 78% and 81%, respectively, of infants born before 34 GW compared to 57% and 34%, respectively, of infants born after 34 GW. The distribution of plasma melatonin concentrations was found to be correlated with gestational age at both time-points (*p* < 0.001). Median urine aMT6s concentrations were significantly lower in infants born before 34 GW, both on Day 1 (230 ng/L vs. 533 ng/L, *p* < 0.0001) and on Day 3 (197 ng/L vs. 359 ng/L, *p* < 0.0001). In conclusion, melatonin secretion appears very low in preterm infants, providing the rationale for testing supplemental melatonin as a neuroprotectant in clinical trials.

## 1. Introduction

Melatonin is the principal hormone secreted by the pineal gland, and its rhythmic secretion is induced by the light/dark cycle and inhibited by artificial light [1,2,3]. Melatonin also has antioxidant, anti-inflammatory and anti-excitotoxic effects, as demonstrated both in rodents and human neonates [4,5,6,7,8,9]. Experimental data strongly support the neuroprotective role of melatonin in animal models of perinatal brain injury [9,10]. Therefore, melatonin appears a promising molecule to prevent brain insults associated with prematurity or birth asphyxia [4,5,6,7,8]. While several studies have quantified melatonin secretion in children, only a few have measured the secretion of melatonin in preterm and term neonates [11,12,13,14]. Studies carried out in preterm infants have yielded inconclusive results: while a decrease in urine aMT6s levels has been reported with gestational age (GA) at birth [11], Kennaway et al. reported reduced 6-sulfatoxy-melatonin (aMT6s) urine concentrations during the first three months after birth in preterm infants compared to term infants [15]. Other studies also reported higher plasma melatonin levels in term infants compared to preterm neonates [12,13]. The variability of melatonin levels observed in preterm infants is mostly related to three factors: the luminosity of the environment, the sensitivity and specificity of assays used on different biological samples [1,2], and drug interactions [16].

We hypothesized that preterm delivery would be associated with lower melatonin levels compared to full-term delivery. To test this hypothesis, we prospectively recruited a large cohort of infants to simultaneously measure melatonin concentrations in plasma and urine of preterm and term infants, and in maternal milk.

## 2. Results

### 2.1. Baseline Characteristics of Patients

A total of 209 infants were recruited from 169 mothers, stratified into two groups according to GA (24^0/7^–33^6/7^ GW, *n* = 110 and 34^0/7^–41^6/7^ GW, *n* = 99, Supplemental Figure S1).

The main baseline characteristics for both GA groups are shown in Supplemental Table S1.

For both GA groups, sampling for plasma melatonin, urine aMT6s and milk melatonin is described in Supplemental Table S2.

### 2.2. Plasma Melatonin Concentrations

**Figure 1:**
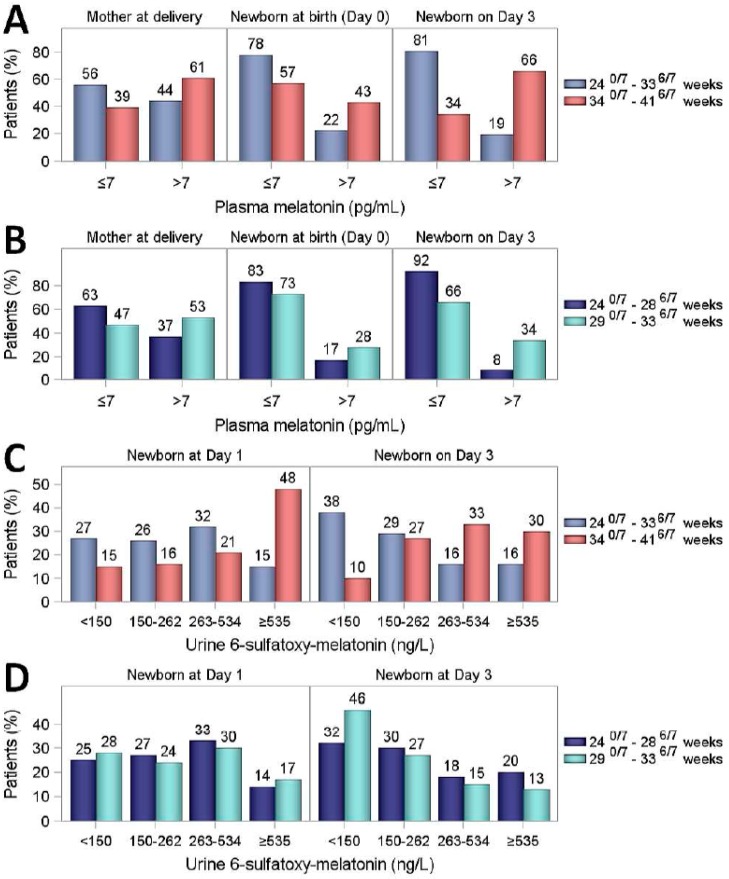
Distribution of plasma melatonin and urine a6MTs concentrations. (**A**) Plasma melatonin concentrations (pg/mL) in mothers at delivery, and the overall population recruited, split into 2 concentrations ranges (≤7 pg/mL, threshold of detectability, and >7 pg/mL). (**B**) Plasma melatonin concentrations (pg/mL) in infants born between 24^0/7^ and 33^6/7^ gestational weeks (GW), split into 2 groups (24^0/7^–28^6/7^ GW and 29^0/7^–33^6/7^ GW). (**C**) Urine a6MTs concentrations (ng/L) in the overall population. (**D**) Urine a6MTs concentrations (ng/L) in infants born between 24^0/7^ and 33^6/7^ GW, split into 2 groups (24^0/7^–28^6/7^ GW and 29^0/7^–33^6/7^ GW).

**Figure 2:**
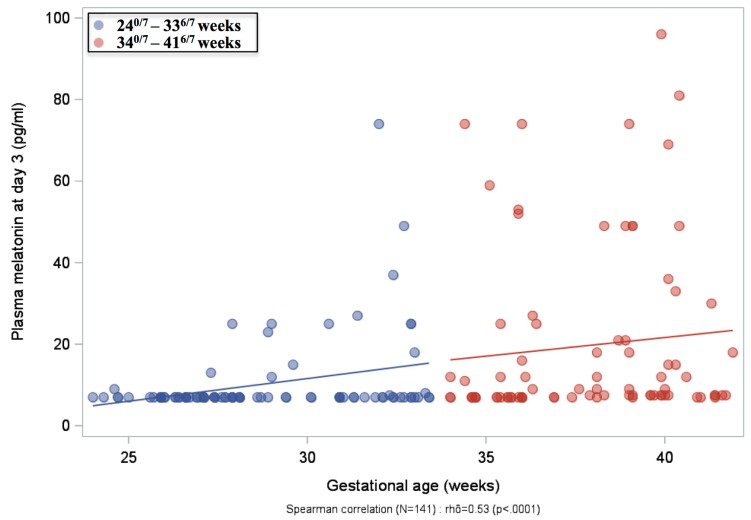
Spearman correlation between plasma melatonin concentration (pg/mL) and gestational age on Day 3.

Plasma melatonin concentrations were measured in 152 mothers at delivery, 167 infants at birth and 173 infants on Day 3 in both GA groups (Table 1). In mothers, median plasma melatonin concentrations were significantly lower when delivery occurred before 34 GW compared to those after 34 GW (*p* = 0.02). Accordingly, we found significantly lower plasma melatonin concentrations in infants born before 34 GW both at birth (*p* = 0.002) and on Day 3 (*p* < 0.0001), compared to infants born after 34 GW. In infants born before 34 GW, longitudinal plasma melatonin measurements after birth showed low values at subsequent time points (Days 10, 25 and 55) without any postnatal increase (Table 1). Distribution of plasma melatonin concentrations is depicted in Figure 1A. A large majority of infants born between 24^0/7^ and 33^6/7^ GW were considered to be melatonin deficient (plasma melatonin concentrations ≤7 pg/mL, measured in 78% at birth and 81% on Day 3). In contrast, only 57% and 34% of infants born between 34–41 GW were found to be deficient at birth and on Day 3, respectively. Plasma melatonin concentrations were highly significantly different between the two GA groups (*p* < 0.0001, both at birth and on Day 3, using a Cochran–Armitage test for ordinal data). No significant difference in plasma melatonin concentrations was observed within the lowest GA group between extremely preterm infants (24–28 weeks) and very preterm infants (29–33 weeks) (Figure 1B). However, when all plasma melatonin concentrations (*n* = 141) on Day 3 were correlated with GA, the Spearman correlation coefficient of 0.53 was found to be highly significant (*p* < 0.0001, Figure 2). Plasma melatonin concentrations in mothers and newborns at birth and on Day 3 were not significantly dependent on the time of sampling (Supplemental Table S3). 

### 2.3. Urine aMT6s Excretion

Median urine aMT6s concentrations in 24 h samples were significantly lower in infants born between 24^0/7^ and 33^6/7^ GW than in those born between 34^0/7^ and 41^6/7^ GW, both on Day 1 (230 ng/L vs. 533 ng/L) and on Day 3 (197 ng/L vs. 359 ng/L, *p* = 0.0001), Table 2. These significant differences were also observed in both diurnal and nocturnal samples. The distribution of urine aMT6s concentrations is shown in Figure 1C,D. Both on Day 1 and on Day 3, infants born before 34 GW displayed a significantly lower aMT6s concentration (Figure 1C; *p* = 0.0002, *p* < 0.0001, respectively). No significant differences were detected between infants born at 24–28 GW and those born at 29–33 GW (Figure 1D).

### 2.4. Milk Melatonin Concentrations

Median milk melatonin concentrations measured on Day 3 were significantly higher in mothers who delivered before 34 GW compared to those who delivered after 34 GW (20 pg/mL vs. 8 pg/mL, *p* = 0.01, Supplemental Table S4). In mothers who delivered between 24^0/7^ and 33^6/7^ GW, median milk melatonin concentrations were stable, between 16 and 20 pg/mL, from Day 3 to Day 55. 

### 2.5. Multivariate Analyses of Factors Associated with Melatonin Deficiency 

Supplemental Table S5 reports bivariate analyses between plasma melatonin and urine aMT6s concentrations and perinatal variables. Variables with a *p*-value < 0.20 were used for the multivariate analyses shown in Table 3. Low plasma melatonin concentrations in neonates at birth were significantly associated with low plasma melatonin concentrations in the mother (*p* = 0.02), daytime sampling (*p* = 0.03) and epidural analgesia (*p* = 0.04). Low plasma melatonin concentrations in neonates on Day 3 were significantly associated with the group of infants born before 34 GW (*p* < 0.0001) and multiple gestation (*p* = 0.002). Similarly, low urine aMT6s concentrations on Day 1 were significantly associated with the group of infants born before 34 GW (*p* < 0.0001).

Altogether, these data strongly support an association between low GA at birth and low melatonin concentrations during the early neonatal period. 

## 3. Discussion

This prospective multicenter study found that plasma melatonin and urine aMT6s concentrations measured at birth and Day 3 in infants born before 34 GW were lower compared to those measured in more mature infants. In contrast, milk melatonin concentrations were higher in the group of mothers who delivered before 34 GW.

We found that maternal melatonin concentrations were significantly higher when delivery occurred after 34 GW. This is consistent with previous studies showing that maternal blood levels of melatonin progressively increase after 32 GW, especially at night [14,16]. The mechanisms underlying this rise in plasma melatonin with GA remain unclear. It has been proposed that the expression of maternal melatonin and its receptors, MT1 and MT2, by the pineal gland and by the placenta progressively increase during pregnancy [17], partly explaining the similar increase with GA observed in neonates at birth, as shown here by multivariate analyses. However, neonatal melatonin concentrations at later time-points after birth are unlikely to be related to maternal or placental production only, considering the short half-life of melatonin.

The urine metabolite aMT6s has been shown to be a reliable marker of melatonin production in adult humans [18]. Consistent with the lower plasma levels reported in the most immature infants, urine aMT6s excretion was lower from birth to discharge in very preterm infants compared to more mature neonates. These findings are in contrast to those reported by Commentz et al. showing elevated urine aMT6s levels during the first postnatal week in 26 preterm infants born between 26–32 GW, compared to 38 infants born after 33 GW [11]. However, perinatal variables, unit environment and the health status of the neonates recruited in this study, reported 20 years ago, are unknown and likely quite different from current standards including developmental care.

Another intriguing finding of the MELIP study is that urine aMT6s levels measured at term equivalent of age in former preterm infants did not reach those measured in full-term infants at birth. This is in agreement with the report by Kennaway et al., showing that the progressive increase in aMT6s levels usually observed during the first 3 months in full-term neonates is delayed until 8–9 months of corrected age in preterm infants born between 29–35 GW [15]. We found no difference in aMT6s urine excretion between daytime and nighttime suggesting that circadian rhythmic release may appear later. This is in agreement with Kennaway et al. who also reported that the rhythmic secretion of melatonin by the pineal gland is only apparent after 3 months in full-term infants, and delayed by about 9 weeks in premature infants [15]. These authors hypothesized that both environment and prematurity could play a role in the abnormal maturation of brain areas responsible for circadian rhythms during the first weeks of life [18].

In addition to the environment, several factors could interfere with melatonin production and excretion. Light exposure in the NICU and during phototherapy could account for the lower melatonin production observed in the most immature and sickest infants. As melatonin metabolism is mostly mediated by cytochrome P450 (CYP1A) enzymes, interactions between melatonin and other drugs, notably caffeine, are also possible [16].

Experimental studies strongly emphasize the potently neuroprotective capacity of melatonin, regardless of the animal species used, following several types of brain insults at various developmental stages. They also support the feasibility of melatonin use, which has a remarkable safety profile both in animals and humans [5,6,8,19,20,21,22,23,24,25]. However, the dosage of melatonin to be used as a neuroprotectant in preterm infants remains debatable. To date, high-dose melatonin treatment has been reported to be beneficial in the treatment of sepsis and chronic lung disease [5,6].

Finally, we reported here the detection of significantly higher melatonin concentrations in the mother’s milk when delivery occurs very preterm, a difference that was not previously observed by Katzer et al. [26]. Human milk is now clearly recognized as being associated with a better neurological outcome in very preterm infants. However, melatonin provided through breastfeeding is likely insufficient to be neuroprotective per se.

A strength of the MELIP study is the number of infants prospectively investigated, the largest reported in the literature to our knowledge, and recruitment within the two different GA groups, from 24 GW to full-term, was well balanced by our study design. The perinatal period was studied by comparing melatonin levels in both mothers and their neonates. Despite multicenter recruitment, environmental light and phototherapy guidelines were standardized and all information carefully recorded. 

The main limitation of our study is the threshold of detectability of melatonin, restricted by the low blood volume collected in serial sampling, due to ethical considerations (0.1 mL). A substantial proportion of our data are likely overestimated because measurements below the threshold of 7 pg/mL were all reported as equal to 7 pg/mL, suggesting that statistical differences observed between groups may be underestimated in our study. Recently, van Fassen et al. illustrated the added value of accurate and sensitive mass spectrometry-based methods for the analysis of melatonin in plasma [27].

## 4. Methods

### 4.1. Study Design

The MELIP study was a prospective, longitudinal, multicenter study. We enrolled mothers and their neonates in three French perinatal tertiary-care centers between April 2011 and January 2013. Mothers were eligible if they were more than 18 years old, and if they had no known chronic disease or drug therapy (beta-blockers, exogenous melatonin, cimetidine, quinolone).

Eligible neonates were born and delivered between 24^0/7^ and 41^6/7^ gestational weeks (GW). Infants with congenital malformations or known chromosomal aberrations were excluded.

According to GA at birth, infants were separated into two groups (24^0/7^–33^6/7^ GW and 34^0/7^–41^6/7^ GW) with different sample collection time-points.

Trial Registration: clinicaltrials.gov identifier: NCT01340417.

Patient consent: Written informed consent was obtained from all parents of eligible infants.

Ethic approval: The trial was approved by the National Ethics Committee (Comité de Protection des Personnes (CPP) Ile-de-France II, Necker), and by the French data protection authority, the Commission Nationale de l’Informatique et des Libertés (CNIL). The trial was registered at clinicaltrials.gov (NCT01340417) before the first patient was enrolled.

### 4.2. Blood Samples for Melatonin Measurement

Blood samples were collected from the mother and the umbilical vein cord at birth. For infants born between 24^0/7^ and 33^6/7^ GW, blood sampling was repeated on Days 3, 10, 25 and 55 between 08:00 h and 12:00 h. For infants born between 34^0/7^ and 41^6/7^ GW, blood samples were collected during the newborn screening at 72 h of life (Day 3), depending on the time of birth, so that plasma melatonin concentrations were measured at different times of the day and the night, along the nycthemeron.

### 4.3. Urine Samples for aMT6s Measurement

Daytime and nighttime levels of urine aMT6s were measured at different time-points following admission to the NICU or maternity ward (both GA groups: Days 1 and 3; 24^0/7^–33^6/7^ GW group only: Days 10, 25 and 55). Urine samples were collected 24 h a day and analyzed over 24 h and 12 h time periods (daytime and nighttime). 

### 4.4. Collection of Breast Milk Samples

Mother’s milk was collected after electric-pump or hand expression. Five milliliters were obtained at 08:00 h on Days 3, 10, 25 and 55 when delivery occurred between 24^0/7^ and 33^6/7^ GW and on Day 3 when delivery occurred between 34^0/7^ and 41^6/7^ GW. 

### 4.5. Melatonin and aMT6s Assays

Blood was centrifuged at 3000 g for 5 min, and then the plasma was separated and frozen at −40 °C until analysis. Urine samples were kept at 4 °C until the end of collection, then aliquoted and stored at −20 °C until assayed. 

Melatonin measurements were performed by radioimmunoassay according to Claustrat et al. [28]. Briefly, biological samples were previously extracted with diethyl–ether before radioimmunoassay. The detection limit, defined as the concentration corresponding to a displacement of three standard deviations of counts from maximum binding (zero concentration), varied according to the sample volume. It was routinely 7 pg/mL when only 0.1 mL of plasma was available. 

For milk melatonin assays, the standard curve was obtained by dilution in a buffer solution including melatonin-free cow’s milk previously treated with charcoal in order to chelate endogenous melatonin. The dilution linearity test was validated by dilutions of a milk sample with high melatonin concentration. Our melatonin assay meets pathophysiological criteria of validity established over 30 years, especially very low or undetectable melatonin levels in plasma of normal adults sampled during the daytime and in plasma of pinealectomized patients or rat sampled at night.

Urine aMT6s levels were determined by radioimmunoassay as previously reported [29]. Results are expressed in term of quantity/time span (ng/L/12 h).

### 4.6. Main Outcomes and Sample Size Determination

The two main primary outcomes were the comparisons of plasma melatonin and urine aMT6s concentrations between newborns according to their GA at birth. Plasma melatonin deficiency was defined as a concentration below 7 pg/mL. All plasma concentrations below this threshold were considered below or equal to 7 pg/mL (≤7 pg/mL). Milk melatonin concentration was reported as a secondary outcome.

Sample size was based on differences in expected plasma melatonin concentrations in newborns according to their GA at birth. It was calculated with nQuery Advisor 6.01 software assuming that plasma melatonin deficiency would occur in more than 50% of very preterm infants born before 33^6/7^ GW and in only 25% of those born after 34^0/7^ GW. The level was set at 5% and the power at 90%. Based on these assumptions, 77 newborns had to be included in each gestational group. To ensure a well-balanced number of patients from 24 GW to term, similar numbers of infants born at 24–28, 28–33, 34–36 and 37–41 GW were recruited. To account for potential dropouts, technical failures and the potential heterogeneity of patients, a larger total sample size (100 newborns per GA group) was required.

### 4.7. Statistical Analysis 

Non-Gaussian continuous variables were described by the median (interquartile range (IQR): 1st quartile–3rd quartile), minimum and maximum. Categorical variables were described by numbers and percentages. 

Factors associated with plasma melatonin deficiency measured at birth and on Day 3 were studied using 2 logistic models. Factors associated with urine aMT6s concentrations on Day 1 were analyzed using a linear mixed model for repeated data, as concentrations were measured twice. For both models, missing values for outcomes and covariables were replaced using a multiple imputation procedure by fully conditional specification. 

For each outcome, significant covariables at a *p*-value < 0.20 in bivariate analysis were selected for the multivariate analysis. A stepwise selection method estimated the final models containing only significant covariables at a *p*-value < 0.05.

Odds ratios for logistic models or beta coefficients for linear mixed models and their 95% confidence intervals were calculated. All statistical tests were two-tailed with the significance level set at 5%. The analyses were conducted using SAS software (v9.4, SAS Institute Inc., Cary, NC, USA).

In conclusion, this prospective cohort study shows that melatonin production and excretion are significantly lower in preterm infants born before 34 GW when compared to more mature infants. Based on intrinsic properties of this molecule, our data further support the rationale for giving supplemental melatonin as a potential neuroprotectant to this high-risk population. 

## Figures and Tables

**Table 1:** Plasma melatonin concentrations (in pg/mL) in the mother at delivery and in newborns at birth and on Days 3, 10, 25 and 55, compared between the two gestational age groups (24^0/7^–33^6/7^ GW and 34^0/7^–41^6/7^ GW).

Sample	24^0/7^–33^6/7^ Weeks	34^0/7^–41^6/7^ Weeks	*p*-Value *
Mother at delivery Median (IQR) Min; Max	*n* = 77 7 (7–20) 7; 213	*n* = 75 11 (7–50) 7; 158	0.02
Newborn at birth Median (IQR) Min; Max	*n* = 81 7 (7–7) 7; 83	*n* = 86 7 (7–24) 7; 184	0.002
Newborn on Day 3 Median (IQR) Min; Max	*n* = 90 7 (7–7) 7; 74	*n* = 83 8 (7–21) 7; 96	<0.0001
Newborn on Day 10 Median (IQR) Min; Max	*n* = 85 7 (7–7) 7; 74	-	-
Newborn on Day 25 Median (IQR) Min; Max	*n* = 73 7 (7–7) 7; 25	-	-
Newborn on Day 55 Median (IQR) Min; Max	*n* = 47 7 (7–7) 7; 38	-	-

* Wilcoxon-Mann-Whitney test. IQR denotes interquartile range (Q1–Q3).

**Table 2:** Urine 6-sulfatoxymelatonin concentrations (in ng/L) in newborns on Days 1, 3, 10, 25 and 55, compared between the 2 gestational age groups (24^0/7^–33^6/7^ GW and 34^0/7^–41^6/7^ GW).

Sample	24^0/7^–33^6/7^ Weeks	34^0/7^–41^6/7^ Weeks	*p* Value *
**Day 1**			
24 h Median (IQR) Min; Max	*n* = 97 230 (137–425) 48; 1917	*n* = 62 533 (241–830) 48; 4876	<0.0001
Daytime (08:00–19:59) Median (IQR) Min; Max	*n* = 84 192 (103–361) 22; 2358	*n* = 46 538 (195–828) 48; 4458	<0.0001
Nighttime (20:00–07:59) Median (IQR) Min; Max	*n* = 87 222 (124–456) 48; 1836	*n* = 53 413 (210–745) 48; 5294	0.002
**Day 3**			
24 h Median (IQR) Min; Max	*n* = 104 197 (114–336) 16; 4302	*n* = 63 359 (211–647) 48; 6527	0.0001
Daytime (08:00–19:59) Median (IQR) Min; Max	*n* = 93 187 (119;401) 16; 5862	*n* = 49 329 (172;612) 48; 6641	0.003
Nighttime (20:00–07:59) Median (IQR) Min; Max	*n* = 94 178 (112–298) 48; 4252	*n* = 50 350 (204–495) 48; 9469	0.0003
**Day 10**			
24 h urine excretion Median (IQR) Min; Max	*n* = 92 168 (96–253) 47; 1167	-	-
Daytime (08:00–19:59) Median (IQR) Min; Max	*n* = 91 168 (95–259) 48; 1597	-	-
Nighttime (20:00–07:59) Median (IQR) Min; Max	*n* = 81 145 (94–247) 24; 944	-	-
**Day 25**			
24 h Median (IQR) Min; Max	*n* = 82 160 (119–217) 48; 2749	-	-
Daytime (08:00–19:59) Median (IQR) Min; Max	*n* = 75 170 (118–243) 27; 2691	-	-
Nighttime (20:00–07:59) Median (IQR) Min; Max	*n* = 76 147 (93–223) 48; 2807	-	-
**Day 55**			
24 h Median (IQR) Min; Max	*n* = 49 168 (119–257) 53; 1481	-	-
Daytime (08:00–19:59) Median (IQR) Min; Max	*n* = 49 200 (118–249) 53; 1713	-	-
Nighttime (20:00–07:59) Median (IQR) Min; Max	*n* = 45 133 (80–275) 48; 1249	-	-

* Wilcoxon–Mann–Whitney test. IQR denotes interquartile range (Q1–Q3).

**Table 3:** Multivariate analyses of factors associated with plasma melatonin deficiency at birth and on Day 3 and urine aMT6s on Day 1.

Variable	Odds Ratio [IC95%]	*p* Value
**Plasma melatonin at birth ≤7 pg/mL**		
Nighttime sampling (00:00–05:59)	0.41 [0.18; 0.90]	0.03
Plasma melatonin concentration ≤7 pg/mL in mother at birth	0.46 [0.24; 0.87]	0.02
Epidural analgesia	2.13 [1.03; 4.35]	0.04
**Plasma melatonin on Day 3 ≤ 7 pg/mL**		
Lower gestational age group (24^0/7^–33^6/7^ weeks)	5.56 [2.94; 11.11]	<0.0001
Multiple gestation	2.94 [1.47; 5.88]	0.002
**Urine aMT6s on Day 1**		
Lower gestational age group (24^0/7^–33^6/7^ weeks)	2.08 [1.59; 2.70]	<0.0001

Significant variables at a *p*-value < 0.20 in bivariate analyses (see Supplemental Table S5) were entered and retained at the 0.05 level after stepwise selection.

## Supplementary Materials

Supplementary materials can be found at https://www.mdpi.com/1422-0067/20/9/2077/s1.
