# Supplementary material for: Melatonin Levels in Preterm and Term Infants and Their Mothers
Source: Int J Mol Sci. 2019 Apr 27;20(9):2077. doi: 10.3390/ijms20092077 (PMC6540351; doi:10.3390/ijms20092077)
Supplement: Supplementary file 1 [file ijms-20-02077-s001.pdf]

Supplemental Figures and Tables

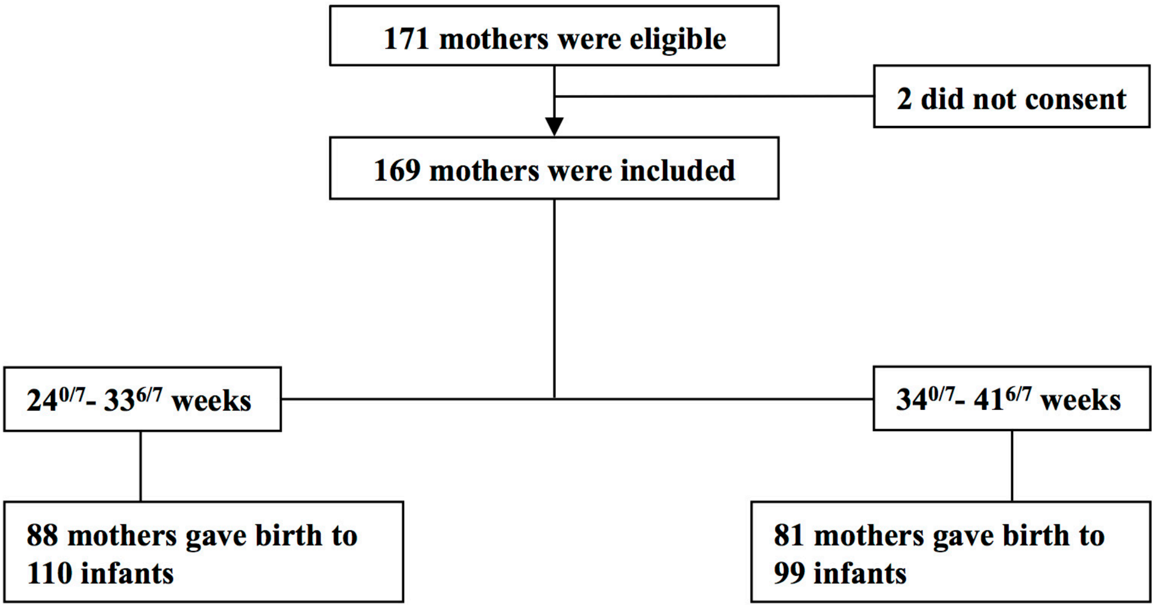

Supplemental Figure S1: Flowchart of patient recruitment in the two gestational age groups ($24^{0/7}$- $33^{6/7}$ weeks and $34^{0/7}$- $41^{6/7}$ weeks).

Supplemental Table S1: Baseline characteristics of infants and their mothers according to gestational age groups.

Variable	Gestational age group	
	**$24^{0/7}$- $33^{6/7}$ GW**	**$34^{0/7}$- $41^{6/7}$ GW**
**Mothers**	**N=88**	**N=81**
Race or ethnic group - N (%)		
Caucasian	47 (54%)	29 (39%)
African	36 (41%)	41 (55%)
Asian	2 (2%)	3 (4%)
Other	2 (2%)	2 (3%)
Gestation		
Median (1st quartile – 3rd quartile – IQR)	2 (2 – 4)	2 (2 – 4)
Min ; Max	1 ; 9	1 ; 7
Parity		
Median (IQR)	2 (1 – 3)	2 (1 – 3)
Min ; Max	1 ; 7	1 ; 7
Pre-eclampsia - N (%)	9 (10%)	1 (1%)
Antenatal glucocorticoid treatment - N (%)	73 (89%)	12 (22%)
Vaginal delivery - N (%)	48 (55%)	65 (81%)
Epidural analgesia - N (%)	31 (65%)	58 (89%)
**Infants**	**N=110**	**N=99**
Gestational age - weeks		
Median (IQR)	28·1 (26·6 – 31·3)	37·9 (35·6 – 39·9)
Min ; Max	24·0 ; 33·4	34·0 ; 41·9
Male sex - N (%)	61 (55%)	42 (42%)
Birth weight - g		
Median (IQR)	1128 (820 – 1500)	2615 (2285 – 3340)
Min ; Max	443 ; 2420	1300 ; 4370
Birth head circumference - cm		
Median (IQR)	26·5 (23·5 – 28,5)	33·0 (32·0 – 35·0)
Min ; Max	2.0 ; 34·5	27·5 ; 37·0
Birth length - cm		
Median (IQR)	37·0 (34·0 – 4.0)	47·0 (45·0 – 5.0)
Min ; Max	24·5 ; 46·0	38·0 ; 54·0
Intrauterine growth restriction below 3rd perc.. - N (%)	8 (7%)	7 (7%)
Apgar score at 5 min		
Median (IQR)	9 (8 – 10)	10 (10 – 10)
Min ; Max	0 ; 10	7 ; 10
Multiple birth - N (%)	42 (38%)	36 (36%)
Phototherapy - N (%)	79 (76%)	30 (31%)
Continuous	27 (34%)	13 (43%)
Discontinuous	52 (66%)	17 (57%)

GW denotes gestational weeks; IQR denotes interquartile range (Q1-Q3).

Supplemental Table S2: Number of samples (plasma, urine, milk) in mothers and infants according to gestational age group.

	$24^{0/7}$ – $33^{6/7}$ GW		$34^{0/7}$ – $41^{6/7}$ GW	
**Mothers**	**N=88**		**N=81**	
	Plasma	Milk	Plasma	Milk
Delivery	N=77		N=75	
Day 3		N=37		N=61
Day 10		N=32		
Day 25		N=20		
Day 55		N=10		
**Newborn**	**N=110**		**N=99**	
	Plasma	Urine	Plasma	Urine
Birth	N=81		N=86	
Day 1		N=97		N=62
Day 3	N=90	N=104	N=83	N=63
Day 10	N=85	N=92		
Day 25	N=73	N=82		
Day 55	N=47	N=49		

GW denotes gestational weeks .

Supplemental Table S3: Comparison of plasma melatonin concentrations (in pg/mL) in mother, and newborn at birth (Day 0) between the 2 gestational age groups and according to delivery time.

Sample	$24^{0/7}$- $33^{6/7}$ GW	$34^{0/7}$- $41^{6/7}$ GW	P-value*
**Mother at delivery**			
Total	N=77	N=75	.02
Median (IQR)	7 (7 – 20)	11 (7 – 50)	
Min ; Max	7 ; 213	7 ; 158	
Nighttime (00:00 – 07:59)	N=14	N=17	.32
Median (IQR)	7 (7 – 42)	11 (7 – 72)	
Min ; Max	7 ; 190	7 ; 158	
Daytime (08:00 – 23:59)	N=60	N=58	.07
Median (IQR)	7 (7 – 19)	11 (7 – 36)	
Min ; Max	7 ; 213	7 ; 154	
**Newborn at birth (day 0)**			
Total	N=81	N=86	.02
Median (IQR)	7 (7 – 7)	7 (7 – 24)	
Min ; Max	7 ; 83	7 ; 184	
Nighttime (00:00 – 07:59)	N=15	N=22	.07
Median (IQR)	7 (7 – 15)	27 (7 – 73)	
Min ; Max	7 ; 58	7 ; 184	
Daytime (08:00 – 23:59)	N=66	N=64	.11
Median (IQR)	7 (7 – 7)	7 (7 – 11)	
Min ; Max	7 ; 83	7 ; 83	

* Wilcoxon-Mann-Whitney test. GW denotes gestational weeks; IQR denotes interquartile range.

Supplemental Table S4: Milk melatonin concentrations (in pg/mL) on Day 3 in the mothers of both gestational age groups and on Days 10, 25 and 55 in mothers who delivered at $24^{0/7}$- $33^{6/7}$ weeks.

Time of sampling	$24^{0/7}$- $33^{6/7}$ GW	$34^{0/7}$- $41^{6/7}$ GW	P-value*

**Day 3**	N=22	N=53	
Median (IQR)	20 (7 – 33)	8 (7 – 16)	.01
Min ; Max	7 ; 137	7 ; 98	
**Day 10**	N=21		
Median (IQR)	16 (7 – 25)	-	-
Min ; Max	7 ; 93		
**Day 25**	N=9		
Median (IQR)	19 (7 – 22)	-	-
Min ; Max	7 ; 48		
**Day 55**	N=4		
Median (IQR)	20 (15 – 28)	-	-
Min ; Max	15 ; 31		

* Wilcoxon-Mann-Whitney test. GW denotes gestational weeks; IQR denotes interquartile range (Q1-Q3).

Supplemental Table S5: Univariate analysis of plasma melatonin and urine 6-sulfatoxy-melatonin concentrations in neonates according to perinatal variable.

**Sample**	**Serum Melatonin**		**Urine 6-sulfatoxy-melatonin**
**Variable**	**Birth**	**Day 3**	**Day 1**
Lower gestational age group ($24^{0/7}$ – $33^{6/7}$ weeks) – P value	.14	<.001	<.001
Male sex – P-value	NS	NS	NS
Multiple gestation – P-value	NS	.003	NS
Plasma Melatonin concentration ≤7 pg/mL in mother at delivery – P-value	.03	.17	.10
Plasma Melatonin concentration ≤7 pg/mL in newborn at birth – P-value	-	NS	NS
Pre-eclampsia – P-value	NS	.04	NS
Intrauterine growth restriction <3 perc. – P-value	NS	NS	NS
Phototherapy – P-value	NS	<.001	.08
Vaginal delivery – P-value	NS	.09	.19
Epidural analgesia – P-value	.09	NS	NS
Serum sampling timing (00:00-05:59) – P-value	.04	NS	-
Urinary sampling timing (20:00-07:59) – P-value	-	<.001	NS

NS denotes not statistically significant.
